# A strong relationship between respiratory variations in pulse pressure (PPV) and airway pressure in fluid nonresponders: a potential explanation for false positive PPV values

**DOI:** 10.1186/cc9472

**Published:** 2011-03-11

**Authors:** LO Hoiseth

**Affiliations:** 1 Oslo University Hospital, Oslo, Norway

## Introduction

Respiratory variations in pulse pressure (PPV) during mechanical ventilation predict fluid responsiveness when the tidal volume is >8 ml/kg [1]. The effect of airway pressure on the ability of PPV to predict fluid responsiveness is less explored. In patients undergoing major abdominal surgery, we found low specificity of PPV and therefore explored the relation between peak airway pressure (Paw) and PPV in fluid challenge nonresponders.

## Methods

Twenty-five patients scheduled for open abdominal surgery with volume controlled ventilation 8 ml/kg, I:E ratio 1:2 and PEEP 5 cmH_2_O were included. Fluid challenges of 250 ml colloid were administered at the discretion of the anesthesiologist. PPV, hemodynamic variables, Paw and stroke volume (SV) measured by oesophageal Doppler were recorded before and after fluid challenges. Responders were defined by an increase in SV >15%.

## Results

**Figure 1:**
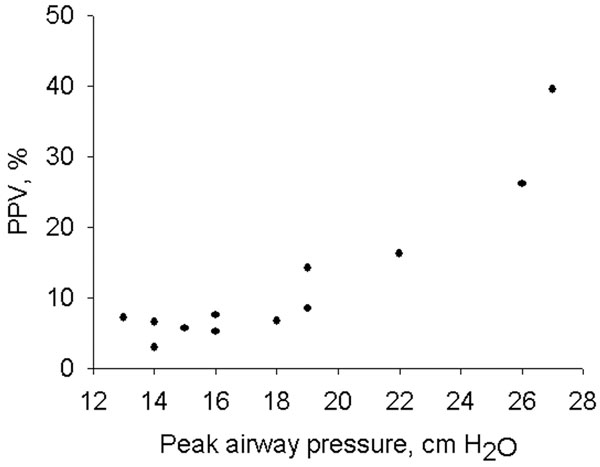
**PPV versus peak airway pressure before fluid challenge in nonresponders**.

Thirty-four fluid challenges were performed. Further data are from analysis of nonresponders; 12 fluid challenges in 11 patients. Specificity of PPV was 0.67. By fluid challenge, PPV was reduced from 7.4 (6.2 to 15.2)% to 6.0 (4.4 to 9.8)% (median, 25th to 75th percentiles), whereas Paw and SV were unchanged. Before fluid challenge, Paw was significantly correlated with PPV (*r *= 0.91, *P *< 0.001) (Figure 1).

## Conclusions

In this study on patients undergoing open abdominal surgery ventilated with 8 ml/kg, specificity of PPV was low. Paw and PPV were strongly correlated and false positive PPVs were associated with high Paw. This finding indicates that not only tidal volume, but also airway pressures may affect the ability of PPV to predict fluid responsiveness.
